# Liver Injury in Patients with COVID-19 without Underlying Liver Disease

**DOI:** 10.3390/jcm11020308

**Published:** 2022-01-08

**Authors:** Monika Pazgan-Simon, Sylwia Serafińska, Michał Kukla, Marta Kucharska, Jolanta Zuwała-Jagiełło, Iwona Buczyńska, Kamila Zielińska, Krzysztof Simon

**Affiliations:** 1 Department of Infectious Disease and Hepatology, Wroclaw Medical University, 50-367 Wroclaw, Poland; sylwia.serafinska@umed.wroc.pl (S.S.); kucharska84m@gmail.com (M.K.); krzysimon@gmail.com (K.S.); 2 Department of Infectious Diseases, Regional Specialistic Hospital, 50-149 Wroclaw, Poland; iwona.buczynska@umed.wroc.pl (I.B.); kamilazielinska123@gmail.com (K.Z.); 3 Department of Internal Medicine and Geriatrics, Faculty of Medicine, Jagiellonian University Medical College, 31-008 Kraków, Poland; michal.kukla@uj.edu.pl; 4 Department of Endoscopy, University Hospital in Kraków, 30-688 Kraków, Poland; 5 Department of Pharmaceutical Biochemistry, Wroclaw Medical University, 50-556 Wroclaw, Poland; jolanta.zuwala-jagiello@umed.wroc.pl

**Keywords:** SARS-CoV-2 infection, liver injury, cholestasis

## Abstract

SARS-CoV-2 shows a high affinity for the ACE-2 receptor, present on the epithelial cells of the upper and lower respiratory tract, within the intestine, kidneys, heart, testes, biliary epithelium, and—where it is particularly challenging—on vascular endothelial cells. Liver involvement is a rare manifestation of COVID-19. Material and Methods: We reviewed 450 patients admitted due to the fact of SARS-CoV-2 infection (COVID-19) including 88 with liver injury. Based on medical history and previous laboratory test results, we excluded cases of underlying liver disease. The analysis involved a clinical course of COVID-19 in patients without underlying liver disease as well as the type and course of liver injury. Results: Signs and symptoms of liver injury were present in 20% of patients, mostly presenting as a mixed-type pattern of injury with less common cases of standalone hepatocellular (parenchymal) or cholestatic injury. The liver injury symptoms resolved at the end of inpatient treatment in 20% of cases. Sixteen patients died with no cases where liver injury would be deemed a cause of death. Conclusions: (1) Liver injury secondary to COVID-19 was mild, and in in 20%, the signs and symptoms of liver injury resolved by the end of hospitalization. (2) It seems that liver injury in patients with COVID-19 was not associated with a higher risk of mortality. (3) The underlying mechanism of liver injury as well as its sequelae are not fully known. Therefore, caution and further monitoring are advised, especially in patients whose liver function tests have not returned to normal values.

## 1. Introduction

The first case of infection with the new pathogenic beta-coronavirus, SARS-CoV-2, was reported in the Chinese city of Wuhan in December 2019 [1,2]. Unfortunately, the epidemic outbreak could not be confined, unlike the SARS-CoV-1 outbreak in 2002/2003 and the MERS epidemic since 2012, so the infection is now a pandemic [3]. The SARS-CoV-2-associated disease, termed COVID-19, manifests mainly, yet not exclusively, as interstitial pneumonia. In some cases—mostly in older patients with multiple comorbidities—it can lead to acute respiratory distress syndrome and death. In patients with COVID-19, the liver is the second most commonly affected organ after the lungs [4].

Initial publications reported COVID-19-related liver injury manifesting as elevated serum aminotransferases and/or cholestatic enzymes activities in 32.6% of patients [5]. Other authors, however, reported this rate to be as high as 50% upon admission and over 75% during hospitalization. Liver injury was associated with increased severity of COVID-19 in patients from China [6]. Furthermore, underlying liver disease, including metabolic disease, was shown to worsen the prognosis in patients with COVID-19 [7], especially those under 60 years of age [8].

The angiotensin-converting enzyme-2 (ACE2) receptor, which enables viral entry into the cell, plays an important role in COVID-19 pathogenesis. The ACE2 receptor is most commonly found in the intestinal mucosa, testes, adipose tissue, and vascular endothelium. It is slightly less abundant in the lungs (the density increases in smokers), but it is also present in the liver, mainly in cholangiocytes [9]. The RNA sequencing studies carried out in patients with COVID-19 demonstrated a significantly higher ACE2 receptor expression in cholangiocytes (59.7%) compared to hepatocytes (2.6%), indicating that SARS-CoV-2 may directly affect the intrahepatic bile duct and, to a lesser extent, hepatocytes. However, a transmembrane serine protease (TMPRSS2) present in hepatocytes facilitates the fusion of SARS-CoV-2 to cell membranes [10].

SARS-CoV-2 has been shown to exert a direct cytopathic effect in pneumocytes. With severe clinical course, COVID-19 causes immune dysregulation leading to extensive release of inflammatory cytokines and activating immunosuppression. Lung damage is accompanied by endotheliitis in various organs with a tendency for intravascular clot formation [11]. The mechanism of liver injury secondary to SARS-CoV-2 infection is extremely complex. SARS-CoV-2 has been shown to upregulate hepatocyte apoptosis, decrease their regenerative capacity, and affect immune disorder [12]. Due to the high density of ACE2 receptors in cholangiocytes, cholestatic liver injury can occur in some patients. Liver injury secondary to COVID-19 may be triggered by pneumonia-associated hypoxia, may be drug induced (e.g., by remdesivir or other antivirals), or may be an aftermath of critical care received at the ICU (intensive care unit). Other factors potentially leading to liver injury include dysbiosis and hepatointestinal damage, vascular lesions (endotheliitis), and right ventricular failure. However, an exacerbation of pre-existing liver disease is also possible. Undoubtedly, all of the abovementioned mechanisms of liver injury secondary to COVID-19 coexist and overlap with each other [13].

In this paper, we present diverse and complex cases of liver injury secondary to a SARS-CoV-2 infection in patients hospitalized at our center in the spring of 2020. Patients underwent oxygen treatment in line with the recommendations developed by the Polish Association of Epidemiologists and Infectiologists. If clinically indicated, they were administered antibiotics (i.e., cephalosporins and macrolides) and low molecular weight heparin. A few patients were administered lopinavir/ritonavir, ribavirin, or chloroquine as a part of clinical trials, while some were treated with tocilizumab and glucocorticosteroids in the ICU setting. During that period, remdesivir, convalescent plasma, or monoclonal antibodies were not used as regular or experimental treatment in our center. Once liver injury was diagnosed, none of the patients received any hepatoprotective treatment.

Aim of the study The aim of this study was to determine the frequency, type, and severity of de novo liver injury seen in patients with COVID-19 and to evaluate the association between liver injury, SARS-CoV-2 eradication time, and COVID-19 severity.

## 2. Materials and Methods

We followed-up 88 patients (out of 450 patients treated at our center for COVID-19 between 6 March 2020 and 10 May 2020) who presented with liver injury secondary to SARS-CoV-2 infection. A COVID-19 diagnosis was confirmed with a positive PCR test. Patients with baseline liver pathology (including hepatitis B infection, hepatitis C infection, autoimmune hepatitis, hepatocellular carcinoma, and chronic inflammation as well as cirrhosis of other etiologies such as alcoholic liver disease, and evident nonalcoholic fatty liver disease (NAFLD) diagnosed with serological tests, lab tests, and medical history) were excluded from the study. Unfortunately, patients were not assessed via abdominal ultrasound. Therefore, we cannot definitely exclude liver steatosis in some patients with overweight or obesity with coexisting metabolic diseases such as type 2 diabetes mellitus or arterial hypertension.

The course of SARS-CoV-2 infection in patients was studied based on clinical presentation and the results of selected laboratory tests. Alongside oxygen saturation (SpO2) measured by 24 h continuous recording, the following laboratory assays were determined: complete blood count (CBC), lymphocyte/neutrophil ratio (LNR), C-reactive protein (CRP), procalcitonin, D-dimers, total bilirubin, aspartate aminotransferase (AST), alanine aminotransferase (ALT), alkaline phosphatase (ALP), gamma-glutamyltransferase (GGGT), and lactate dehydrogenase (LDH). Upper limits of norm (ULN) were as follows: for ALT 55 U/L, for AST 34 U/L, for GGTP 36 U/L, and for ALP 53 U/L. Fasting blood samples were collected for the assays. Based on the presence and severity of the inflammatory response in lungs, they were classified using a four-point scale: mild—where the patient did not need oxygen therapy; moderate—patient needed conventional oxygen therapy; severe—patient needed high-flow nasal cannula oxygen or mechanical ventilation; four—death. Oxygen therapy was required in all patients considered as being in moderate to severe condition. The oxygen demand ranged from 3–5 to 60 L/min (few patients needed high-flow nasal cannula (HFNC)); there were also single cases that required intubation and mechanical ventilation.

We analyzed clinical and laboratory parameters at three time points: baseline (upon first presentation with infection), on day eight of inpatient admission, and on the last day of hospitalization (regardless of its length). Patients were divided into three groups: with parenchymal injury—showing an isolated increase in transaminases activity; with cholestatic injury—showing an isolated increase in GGTP and/or bilirubin activity; with mixed types of injury (Group 3)—showing increased activity of ALT, AST, and inflammatory enzymes, mainly GGTP but also FA and bilirubin.

We assessed comorbidities (listed in Table 1), time to eradication of SARS-CoV-2, and time to resolution of liver injury as well as mortality in all three subgroups.

Sixteen (18%) patients died during hospitalization. Out of those, two were assessed as being in moderate clinical condition, five as moderate to severe, and nine as severe on admission. In line with the regulations in force at that time, all patients were hospitalized until SARS-CoV-2 eradication was confirmed by PCR test.

As a part of the statistical analysis, between-subject comparisons were made using Welch’s *t*-test (for two groups) or ANOVA (for more than two groups). The chi-squared test was used to verify distributions for contingency tables larger than 2 × 2, whereas Fischer’s exact test was used to verify distributions for 2 × 2 contingency tables. Results are presented using descriptive statistics: the minimum, mean and maximum value (tables). Additionally all statistical tests were performed with significance level equal to 0.05 (*p* < 0.05).

## 3. Results

Out of 450 patients admitted with COVID-19, we identified 88 cases of liver injury based on clinical presentation and biochemical markers. These included 51 men at an average age of 63 years old (range: 29–85) and 37 women at an average age of 61 years old (range: 25–90). On admission, 45% of subjects (17 women and 23 men) did not need oxygen therapy. A further 37% of subjects (18 women and 19 men) needed low-flow oxygen therapy, and 10% (two women and eight men) needed immediate HFNC therapy.

Liver injury upon admission was present in 75 patients and developed during hospitalization in the remaining 13 cases (mostly women). Liver injury was shown in patients with (*n* = 54; 62%) and without (*n* = 34; 38%) comorbidities. The basic demographic and clinical parameters of the study group are shown in Table 1.

The age distribution analysis indicated that the age of 60 years was a threshold for severe course of COVID-19 in both the male and female subsets. The patients in the overweight group had an average BMI of 26.34. The average female BMI was 26.21 (19.47–38.8), and the average male BMI was 26.46 (20.94–38.36). It was observed that deaths in the course of COVID-19 in obese patients were not more frequent than in patients with a lower weight, *p* = 0.28. Liver injury occurred in both women and men.

Women below 60 years of age tended to have lower ALT, AST, ALP, and GGT activities than men. Women above 60 years of age developed hepatocellular liver injury at a later stage during hospitalization. However, the only female deaths in our cohort were in this very subgroup.

The most common symptoms of COVID-19 were fever, observed in 80% of patients (32 women and 38 men); cough, observed in 74% of patients (30 women and 35 men); dyspnea, present in more than half of patients, i.e., 56% (22 women and 27 men). The less frequent ones were diarrhea and muscle and joint pain, observed in 19% and 18%, respectively. The rarely observed symptoms were loss of smell and taste, sore throat, and rhinitis, and each was found in 6% of patients, equally frequently in both women and men.

### 3.1. Hepatocellular Liver Injury

In the group with hepatocellular liver injury, 14 women (38%) and 14 men (27%) had elevated ALT and AST levels. The ALT values normalized towards the end of hospitalization in 11 cases from an initial mean level of 80 U/L. The same 11 patients had either normal or mildly elevated AST levels throughout the entire hospitalization.

The ALT and AST values, elevated at baseline, did not normalize until the end of hospitalization in another 26 patients with the same liver injury pattern. In those patients, the mean ALT level on admission was 65 U/L. It increased further to 120 U/L on day eight, reaching 150 U/L at discharge. The AST level on admission was 88 U/L. Although it decreased to 77.75 U/L, the value by the end of hospitalization did not return to the normal range.

#### 3.1.1. Cholestatic Liver Injury

Cholestasis was present in only nine patients (10%) and was more frequent among woman than man (6% of men and 8% of women). The association between sex and cholestatic liver injury was significant (*p* < 0.05). All affected men and most women (all except for one) were at least 60 years old. In this group, we observed normal baseline ALT (29 U/L) and AST (32 U/L) values, and the values remained within the reference range during the hospitalization and at its end. Cholestatic enzyme (i.e., GGT and ALP) and bilirubin levels were consistently elevated throughout the hospitalization (Table 2).

#### 3.1.2. Mixed-Type Liver Injury

Mixed-type liver injury was the predominant type of liver injury secondary to COVID-19 in the study cohort. It was shown in 45 patients (51% of the study group, 16 women and 29 men). The laboratory biomarkers of that subgroup are summarized in Table 3. The elevated parameters normalized towards the end of the inpatient treatment in only three female and three male patients.

Interestingly, there was a significant positive correlation between ALT and CRP levels in this subgroup (*p* = 0.002).

Regardless of the liver injury pattern, the duration of hospitalization for all 62 patients (not counting the 16 deaths) did not differ significantly from that of patients without clinically evident liver disease.

ALT/AST levels returned to normal values in 26% of patients and GGT in 12% of patients (Table 3).

#### 3.1.3. Mortality

Sixteen patients out of the 88 cases of liver injury secondary to COVID-19 (18%, 13 men and three women) died. According to the baseline assessment, this group included two patients in moderate clinical condition, five in moderate to severe clinical condition, and nine in severe clinical condition. Interestingly, all women who died were over 60 years of age. For a comparison, the mortality rate in patients with COVID-19 without liver pathology was 20%. The cause of death in all cases was the progression in lung disease, with zero cases where death would be caused by liver failure.

**Figure 1:**
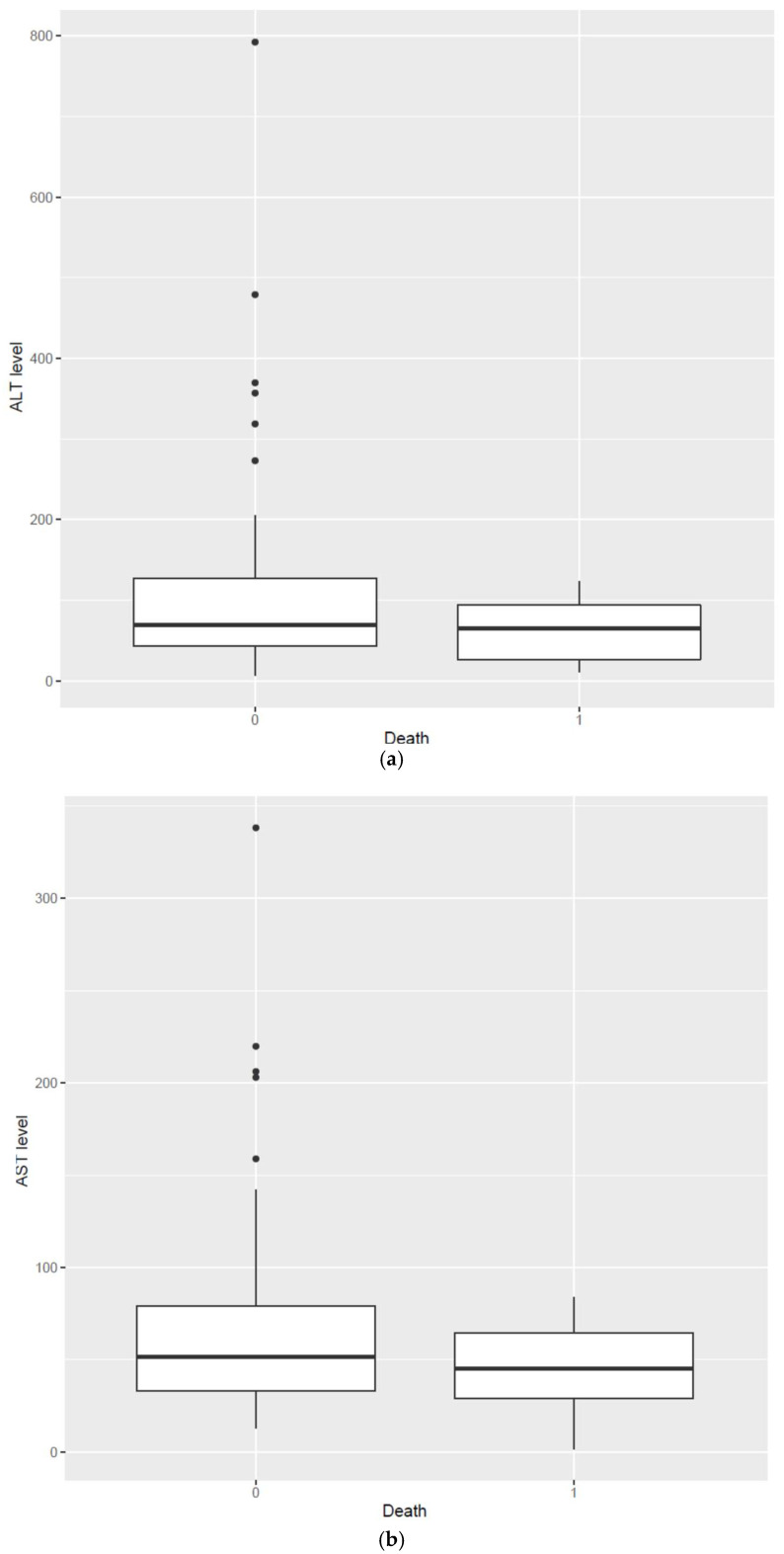
(**a**) ALT activity: second measurement (seven days after admission) in patients with parenchymal and mixed liver injury. First component: COVID-19 survivors. Second component: patients who eventually died of COVID-19. (**b**) AST activity: second measurement (seven days after admission) in patients with parenchymal and mixed liver injury. First component: COVID-19 survivors. Second component: patients who eventually died of COVID-19.

During hospitalization, we observed a progressive increase in AST, ALT, GGT, and ALP levels, which reached very high values (over ten-fold the upper normal range) in those patients who required ICU treatment due to the fact of multiple organ failure and subsequently died (Figure 1) The duration of inpatient treatment did not differ significantly between those who died of COVID-19 and those who were discharged home.

## 4. Discussion

In this study, 88 (20%) of 450 patients hospitalized for COVID-19 presented with liver injury, after those with pre-existing liver pathology were excluded from analysis. This rate is very similar to the results of the meta-analysis by Mao et al., where in 12 studies that covered a total of 1267 patients, abnormal liver function tests were found in 19% of cases [14]. In the meta-analysis by Kulkarni et al., 23.1% of 20,874 patients with COVID-19 were found to have abnormal liver function tests. The prevalence of pre-existing liver disease in the same cohort was only 3.6% [15]. Many authors reported even higher incidence of liver injury/hepatitis in patients with COVID-19, although the rates of pre-existing liver disease in those studies were not provided. A study by Chen et al. showed that 28% of 99 patients with COVID-19 presented with elevated ALT and 35% with elevated AST levels; only one patient developed severe liver function damage [16]. Other authors from China observed elevated AST, ALT, and bilirubin levels in, respectively, 31.6%, 35.4%, and 5.1% of patients hospitalized for COVID-19 who did not require ICU treatment [17]. In contrast, among those admitted to the ICU due to the fact of respiratory failure secondary to COVID-19, up to 60% presented with liver injury [18].

Interestingly, towards the end of hospitalization, we observed a return of once elevated biomarker levels (i.e., GGT, ALP, AST, and ALT) to normal values in 25% of patients, especially those with isolated hepatocellular and cholestatic liver injury. This may indicate the mild nature of liver injury secondary to SARS-CoV-2 infection. This finding is consistent with the observations of other authors [19].

Our observations come from the first wave of COVID-19, when patients did not yet have access to remdesivir. Treatment with remdesivir is often associated with increased levels of AST and ALT and other laboratory biomarkers of liver injury [20].

Despite the proven significantly higher density of ACE2 receptors on cholangiocytes and their negligible presence in hepatocytes, most cases of liver injury in our cohort were hepatocellular (32%) or mixed-type cases (51%); cases of isolated cholestatic liver injury were very rare (7%). Our observations are consistent with results reported by other authors [21].

In our study cohort, there was no significant within-subjects difference between aminotransferase levels at different stages of COVID-19. This is inconsistent with the findings reported by other researchers, who observed a more evident increase in AST than ALT levels in patients with COVID-19-associated liver injury. The mechanisms of this phenomenon are not fully clear, yet they may include a COVID-19-associated mitochondrial dysfunction, SARS-CoV-2-induced hepatic steatosis, and impaired liver perfusion secondary to microvascular thrombosis [22]. In autopsy studies of patients with COVID-19, however, there was no evidence of viral inclusion in liver tissue. What these studies nevertheless demonstrated was microvesicular steatosis, which would suggest a different mechanism of liver injury, such as hypoxia or impaired perfusion, rather than a direct cytopathic effect of the virus [22]. In our cohort, no correlation was found between the baseline AST and ALT levels and the risk of death due to the fact of COVID-19, which some researchers seem to confirm. This observation, however, contradicts the results of other studies, where a positive correlation was demonstrated between ALT levels and levels of such inflammatory markers as CRP, D-dimers, ferritin. and interleukin-6 (Il-6). The Il-6 level, on the other hand, correlates with disease severity [23,24]. Liver inflammation is associated with disturbances in hepatokine synthesis. A recent study has revealed disbalance in some hepatokine levels in COVID-19. These disturbances were associated with disease severity and patients outcome. [25]. Further studies are required to clarify the exact role of hepatokines in liver injury in COVID-19.

In our cohort, we observed a positive correlation between ALT and CRP only in patients with mixed-type liver injury. However, this did not translate into a more severe course of COVID-19 in these patients.

We also observed that the AST, ALT, ALP, and GGT levels did not return to normal values throughout the course of hospitalization in the 16 patients who died of COVID-19. This supports the view that liver injury in these patients was part of multiple organ failure, aggravated by hypoxia or certain drugs such as tocilizumab [26].

Our observations do not confirm previous suggestions that liver injury of any kind is associated with a higher risk of COVID-19 mortality. And there are other authors who seem to agree with us. A study by Yang et al., carried out in 52 critically ill patients treated in an ICU setting for COVID-19 pneumonia, demonstrated that hepatitis was not associated with a higher risk of death. COVID-19-induced hepatitis was demonstrated in 30% of patients who survived and 28% of those who died [27]. However, the patients in our study had no evidence of a pre-existing liver disease, which makes a significant difference. Patients with pre-existing liver disease, in particular cirrhosis, have a higher risk of death compared to those without known liver pathology [28]. The study by Zelenika et al. showed that FibroScan-AST (FAST) score was positively associated with clinical severity and 30 day composite outcome of mechanical ventilation (MV) or death among patients hospitalized due to the fact of COVID-19 [29]. Liver injury may be associated with overweight and obesity. Obesity is strictly associated with liver steatosis, which is a hallmark of NAFLD. Visceral (abdominal) adipose tissue is a very fruitful source of adipokines and cytokines, which influence, for example, inflammatory and metabolic processes. There are some studies that suggest COVID-19 is involved in dysregulation of adipokine production [30]. Our study did not show any differences when comparing patients with a BMI < 25 kg/m^2^ to those with a BMI ≥ 25 kg/m^2^. In our study, the mortality rate in obese patients was not different from the mortality rate in patients with normal weight and overweight. In addition, no differences were observed in liver injury resolution at the end of hospitalization between patients with a normal BMI and patients with a BMI higher than normal.

In summary, the pathomechanism of liver injury secondary to COVID-19 is undoubtedly very complex, and its possible clinical sequelae require further monitoring and research [31].

Our study is by all means limited by the small size of our study group and the fact that the observations came from the first wave of COVID-19 infections, when not all of the currently used drugs were available. Another limitation is that patients’ treatment included neither routine abdominal ultrasound checks nor all biochemical tests that could reveal even minor cases of fatty liver.

## 5. Conclusions

1. Liver injury secondary to COVID-19 is common, presents mainly as a mild hepatocellular or mixed-type injury, and affects patients regardless of their pre-existing liver disease status. In our cohort, signs and symptoms of liver injury were revealed in 20% of patients with COVID-19. Out of those, only 25% experienced a resolution of symptoms during hospitalization due to the fact of SARS-CoV-2 infection;
2. It seems that liver injury in patients with COVID-19 with no underlying liver disease was not associated with higher mortality risk; however, we cannot exclude the coexistence of fatty liver in some of the patients;
3. The underlying mechanism of liver injury as well as its sequelae are not fully known. Therefore, caution and further monitoring are advised, especially in patients whose liver function tests have not returned to normal values.

## Figures and Tables

**Table 1:** Baseline characteristics of the study group.

		Female		Male		Total	
Number of cases		37	minimum/maximum	51	minimum/maximum	88	minimum/maximum
Age	mean	60.9	25/90	63.1	29/85	62.2	25/90
Age	<50	9		7		16	
	50–60	7		9		16	
	60–70	21		11		32	
	>70	14		10		24	
BMI	mean	26.2	19.5/36.8	26.5	20.9/36.3		
Number of cases		37		51		88	
Duration of hospitalization (days)	mean	17.8	0/39	20.8	0/69	19.5	69
Symptom duration (days)	mean	13.2	0/34	15.1	0/43	14.3	0/43
Time to infection eradication (days)	mean	24	8/51	24.5	7/48	24.3	7/51
	not eradicated	7		13		20	
COVID-19 severity							
1		0		1		1	1%
2		17		23		40	44%
3		18		19		37	42%
4		2		8		10	13%
Comorbidities							
Diabetes		5	14%	12	24%	17	19%
Cancer (lung, colorectal, and breast)		7	19%	6	12%	13	15%
Hypertension		16	43%	27	53%	43	49%
Ischemic heart disease		4	11%	4	8%	8	9%
Valvular disease		2	5%	0	0%	2	2%
Artificial heart valve		0	0%	3	6%	3	3%
Stroke		1	3%	1	2%	2	2%
Arrhythmias		4	11%	4	8%	8	9%
Myocardial infarction		0	0%	1	2%	1	1%
Lung cancer		1	3%	1	2%	2	2%
COPD		1	3%	3	6%	4	5%
Pneumothorax		0	0%	1	2%	1	1%
Obstructive sleep apnea		0	0%	1	2%	1	1%
Asthma		1	3%	1	2%	2	2%
Pattern of injury							
Hepatocellular		14	38%	14	27%	28	32%
Cholestatic		3	8%	3	6%	6	7%
Mixed		16	43%	29	57%	45	51%
n/a		4	11%	5	10%	9	10%

**Table 2:** Liver function tests in patients with liver injury upon admission, during hospitalization and, upon discharge.

		Upon Admission				During Hospitalization			Upon Discharge		
		No Liver Injury	Cholestatic Liver Injury	Hepatocellular Liver Injury	Mixed-Pattern Liver Injury	Cholestatic Liver Injury	Hepatocellular Liver Injury	Mixed-Pattern Liver Injury	Cholestatic Liver Injury	Hepatocellular Liver Injury	Mixed-Pattern Liver Injury
ALT	Minimum	12.0	7.0	17.6	19.2	7.0	21.4	35.7	16.0	12.4	10.0
	Mean	20.4	31.1	55.0	86.5	29.5	59.9	101.5	33.0	68.0	61.9
	Maximum	42.1	40.4	107.1	341.0	56.0	369.4	792.0	70.0	134.3	350.0
AST	Minimum	19.7	21.0	27.1	23.9	13.0	14.0	1.6	17.0	25.0	13.1
	Mean	29.4	32.8	52.3	87.0	28.0	44.3	64.9	45.3	50.8	38.0
	Maximum	39.7	44.0	251.0	413.0	57.0	96.0	338.0	72.0	73.0	233.0
GGT	Minimum	17.0	46.6	22.4	46.3	49.5	19.7	47.0	33.6	34.1	44.5
	Mean	26.2	61.0	31.6	134.5	62.5	39.9	120.0	41.2	42.1	78.8
	Maximum	37.7	144.7	36.0	863.0	634.0	320.0	654.0	65.3	87.0	1128.0
ALP	Minimum	39.0	46.0	42.0	39.0	62.0	42.0	48.0	96.0	58.0	49.0
	Mean	63.0	73.0	73.0	89.5	83.5	64.0	98.0	96.0	68.0	95.5
	Maximum	78.0	202.0	133.0	516.0	105.0	111.0	478.0	96.0	78.0	214.0

**Table 3:** Cases with elevated ALT, AST, ALP, and GGT together with parameter normalization towards the end of hospitalization, regardless of liver injury pattern.

	Cases of Hepatitis	Diagnosed upon Admission	Diagnosed during Hospitalization	Hepatitis Resolved upon Discharge	Hepatitis Did Not Resolve	Data on Symptom Resolution Not Available	Resolution Rate in Patients with Hepatitis
Hepatocellular	73	55	16	19	49	5	0.26
Cholestatic	51	45	6	6	39	6	0.12
